# Being Smart Is Not Enough: Personality Traits and Vocational Interests Incrementally Predict Intention, Status and Success of Leaders and Entrepreneurs Beyond Cognitive Ability

**DOI:** 10.3389/fpsyg.2020.00204

**Published:** 2020-02-18

**Authors:** Sabine Bergner

**Affiliations:** Department of Corporate Leadership and Entrepreneurship, University of Graz, Graz, Austria

**Keywords:** Big Five, cognitive ability, entrepreneurship, incremental validity, intelligence, leadership, vocational interest

## Abstract

Three separate studies demonstrate that socio-emotional skills add incremental validity beyond cognitive ability when predicting leadership and entrepreneurship intention, emergence as well as success. Study 1 uses a longitudinal approach and tests the cognitive ability and vocational interests of 231 students to predict their leadership and entrepreneurship intention. It demonstrates that cognitive ability predicts their intention to become a business leader or entrepreneur 2 years in the future. Importantly, the vocational interests “enterprising” and “social” increase this ability-driven prediction of leadership and entrepreneurship intention (Δ*R*^2^_Lead.Intent._ = 15%, Δ*R*^2^_Entre.Intent._ = 9%). Study 2 investigates 123 business leaders and shows that those with higher cognitive ability more likely emerge as top-level leaders, receive more income and get slightly better supervisor-ratings on their performance. The leaders’ Big Five traits (openness, conscientiousness, extraversion, agreeableness, emotional stability) added validity beyond cognitive ability when predicting these criteria (Δ*R*^2^_Income_ = 9%, Δ*R*^2^_Lead.Level_ = 8%, Δ*R*^2^_Perform._ = 15%). Finally, Study 3 includes 155 participants and demonstrates that cognitive ability predicts a person’s entrepreneurial status but not performance. Additionally, considering the Big Five traits improves the prediction of who becomes an entrepreneur and successfully performs as such (Δ*R*^2^_Status_ = 7%, Δ*R*^2^_Perform._ = 18%). Importantly, selected Big Five traits and vocational interests boost the importance of cognitive ability in the field of leadership and entrepreneurship. Concluding, this series of studies suggests that it is the combination of personality traits or interests with cognitive ability which is most powerful when predicting leadership and entrepreneurship intention, emergence and success.

## Introduction

One of the most important predictors of job success is a person’s cognitive ability. Higher cognitive ability leads to more success at work and becomes even more relevant when jobs become intellectually challenging (Salgado et al., 2003). In addition to cognitive ability, socio-emotional skills such as conscientiousness or emotional stability also influence success (He et al., 2019). Socio-emotional skills play a particularly crucial role in so-called *weak* situations in which the degree of freedom for individual action is large and thus success strongly relies on a person’s character (Seibert et al., 1999).

Responsible jobs in business, such as being a leader or entrepreneur, certainly offer both high intellectual challenge and great freedom for action. Here the question arises as to whether both cognitive ability and socio-emotional skills are conjointly needed to become a successful leader and entrepreneur or whether one can compensate the other. Surprisingly, research is relatively silent about this matter. For instance, the conjoined effect of cognitive ability and socio-emotional skills has received scant attention in the field of entrepreneurship. The few existing findings provide promising insights, yet their generalization seems limited mainly due to two reasons. First, they use proxies rather than reliable tests for measuring ability and second, they refer only to a small number of socio-emotional skills. Our investigation aims at closing this research gap. It focuses on leaders and entrepreneurs as they are powerful players in our society and pursues three goals. The first goal is to examine whether socio-emotional skills increase the validity of cognitive ability when predicting leadership and entrepreneurship *intention*. The second goal refers to the question whether socio-emotional skills add incremental validity over cognitive ability when predicting leadership and entrepreneurship *emergence* and *success*. Finally, the third goal addresses the question in how far socio-emotional skills *interact* with cognitive ability. Here, we examine questions such as ‘Can a leader’s cognitive ability buffer his/her reduced socio-emotional skills?’ or ‘Is a certain level of cognitive ability necessary to unleash the potential of an entrepreneur’s socio-emotional skills?’ To this end, the socio-emotional skills which will be studied in this investigation are vocational interests and personality traits.

**FIGURE 1:**
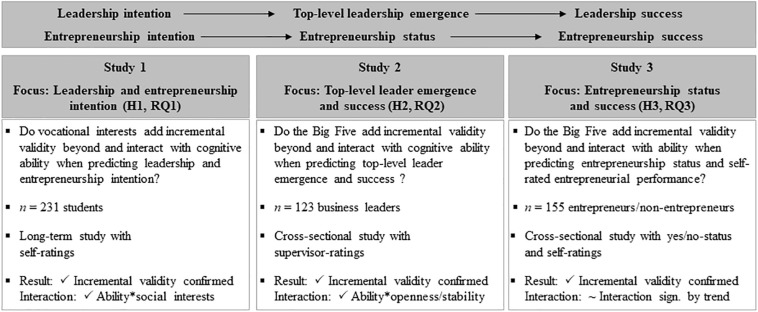
Overview of the three studies within this investigation.

To pursue the three goals, we present three separate studies which are connected on the grounds of the Leader–Trait–Emergence–Effectiveness model (Judge et al., 2009). In line with this model, we see leadership and entrepreneurship intention as the first step toward a career in the field of leadership and entrepreneurship. This intention directly enhances the chance of achieving a leadership or entrepreneurship position which, in turn, is a necessity for achieving leadership and entrepreneurship success (Chan and Drasgow, 2001; Judge et al., 2009). In line with this intention–emergence–success logic, the first study focuses on leadership and entrepreneurship *intention* and summarizes results on whether vocational interests (Holland’s, 1959 RIASEC model) interact with and add incremental validity beyond cognitive ability when predicting them. The subsequent studies emphasis leadership and entrepreneurship *emergence* and *success* as a consequence of expressing the respective intentions. In brief, the second study examines whether personality traits (Five-Factor Model, Costa and McCrae, 1992) interact with and add incremental validity over cognitive ability when predicting leadership emergence and success. Finally, the third study explores whether the same personality traits interact with and add novel information over cognitive ability when predicting a person’s entrepreneurial status and success. Figure 1 provides an overview of the three studies.

This article contributes to existing research in several ways. First, it offers novel insights into the conjoint effect of cognitive ability and socio-emotional skills on a wide range of outcomes relevant for leadership and entrepreneurship. In the field of entrepreneurship, it is among few studies to examine this conjoint effect using a validated ability measure instead of an ability proxy. Second, it enriches research by offering insights into the interplay between cognitive ability and socio-emotional skills. More detailed, it examines whether cognitive ability moderates the impact of socio-emotional skills on leadership and entrepreneurship intention, emergence and success. Finally, it offers information for recruiting, selecting and developing future leaders or entrepreneurs. Subsequently, we discuss relevant research on the importance of cognitive ability, personality traits and vocational interests for leadership and entrepreneurship a) intention, b) emergence and c) success.

### Leadership and Entrepreneurship Intention Is Driven by Cognitive Ability and Interests

The first step toward a successful career in leadership or entrepreneurship is to express intention for such career paths and to actually become a leader or entrepreneur. In fact, intentions are prerequisites of any planned behavior (Ajzen, 1991). Thus, it is not surprising that leadership intention directly predicts a leader’s emergence (Badura et al., 2019) and that entrepreneurship intention directly predicts entrepreneurial status (Kautonen et al., 2015). Consequently, we see leadership intention as a prerequisite for reaching leadership positions and further regard entrepreneurship intention as a prerequisite for entrepreneurial status. How these intentions are influenced by cognitive ability and vocational interests is subsequently summarized.

#### Leadership Intention and Cognitive Ability

People express leadership intention when they show behavior that affects their decision to assume leadership training, roles and responsibilities (Chan and Drasgow, 2001). So far, the impact of ability on leadership intention seems inconclusive. This is particularly true when the focus is set on *cognitive* ability which describes the “ability to understand complex ideas, to adapt effectively to the environment, to learn from experience, to engage in various forms of reasoning, [and] to overcome obstacles by taking thought” (Neisser et al., 1996, p. 77). While studies with impressive longitudinal data or large samples conclude that the intention to lead may not be a simple function of cognitive ability (Chan and Drasgow, 2001; Gottfried et al., 2011; Reichard et al., 2011), recent meta-analytic findings suggest that cognitive ability directly relates to leadership intention (Badura et al., 2019). Despite these somewhat conflicting findings, it is well documented that leading others is a highly complex job, which includes extensive strategic decisions and risk-taking. Because individuals with higher cognitive ability are attracted by this complexity as it fits their ability level, they should also be more likely to gravitate to careers in leadership (Uhl-Bien et al., 2007).

#### Leadership Intention and Vocational Interests

Leading others comes with challenges, which cannot always be met by ability-driven actions. This is particularly true for interpersonal challenges. Showing *interest* in their job helps leaders to stay motivated to conquer difficult situations (Bergner et al., 2019). Among different interests, vocational interests are particularly important, which is why we subsequently focus on them (Holland, 1959).

Vocational interests are most prominently connected to Holland’s (1959) model, which comprises the six interests Realistic, Investigative, Artistic, Social, Enterprising and Conventional. From a theoretical perspective, “enterprising” is the interest most closely related to leadership as enterprising individuals show high interest in leadership tasks. On the one hand, those with strong enterprising interests favor leading groups, negotiating with and convincing others and are thus interested in the *person-driven* tasks of leading. Yet, they also enjoy organizing events, selling ideas and structuring information and are thus also interested in the *data-driven* tasks of leading. Consequently, enterprising interests should enhance a person’s leadership intention, because they are tied to a preference for dealing with both, the person-driven and data-driven leadership tasks (Burke et al., 2006).

Enterprising interests are conceptually closest to social and conventional ones (Holland, 1959), which is why these last two should also influence leadership intention, though to a lesser extent. While individuals with social interests prefer *person-driven* leadership tasks, such as talking to people, giving advice and cooperating with them, individuals with conventional interests rather enjoy *data-driven* leadership tasks, such as checking accounts, checking the observance of guidelines and working with facts and figures. As the three vocational interests enterprising, social and conventional supposedly foster a person’s intention to lead, we will focus solely on them.

#### Entrepreneurship Intention and Cognitive Ability

Those who are determined to set up a new business venture at some point in the future and plan to do so, express entrepreneurship intention (Thompson, 2009). The impact of cognitive ability on entrepreneurship intention seems rather underexplored. However, there is reason to assume that cognitive ability enhances the intent to pursue an entrepreneurial career. First, entrepreneurial jobs are rather complex with less routine and demand strategic thinking and dealing with multifaceted challenges. As previously mentioned, individuals with higher cognitive ability should be drawn to such complexity as it matches their ability level. Second, empirical findings show that skills related to cognitive ability, such as practical intelligence, affect entrepreneurial status (Baum et al., 2011) and it is thus reasonable to assume that cognitive ability also relates to entrepreneurial intention. In the light of these findings, we assume a positive relationship between cognitive ability and entrepreneurship intention.

#### Entrepreneurship Intention and Vocational Interests

Previous research shows that those with higher entrepreneurial interests express more entrepreneurial career prospects and launch their first business earlier (Schmitt-Rodermund, 2004). Using Holland’s (1959) interests, Almeida et al. (2014) further demonstrated that enterprising interests enhance a person’s awareness of entrepreneurial possibilities and also relate to entrepreneurial activities, like selling products. Based on these findings and on the assumption that vocational interests in entrepreneurship are needed to pursue an entrepreneurial career, we presume that Holland’s enterprising interest enhances a person’s entrepreneurship intention. Due to the theoretical proximity of enterprising, social and conventional interests we further assume that social and conventional interests also increase a person’s entrepreneurship intention, although to a lesser extent.

Concluding, cognitive ability and vocational interests ought to drive a person’s entrepreneurship and leadership intention. Importantly, this intention is a prerequisite for actually becoming an entrepreneur or leader which, in turn, is a requirement for achieving entrepreneurial or leadership success (Chan and Drasgow, 2001; Judge et al., 2009). The subsequent sections summarize relevant findings on the link between socio-emotional skills and the *emergence* as well as *success* of leaders and entrepreneurs.

At this point it is vital to keep in mind that cognitive ability most likely drives all three, the intentions, emergence *and* success of leaders or entrepreneurs while vocational interests primarily relate to intentions (Thomas et al., 2001). In fact, vocational interests seem less important for the emergence and success of leaders or entrepreneurs while other socio-emotional skills like personality traits more strongly influence them (e.g., Barrick et al., 2001; Zhao and Seibert, 2006). Hence, we will subsequently focus on personality traits and discuss their role alongside cognitive ability when addressing the impact of socio-emotional skills in leadership and entrepreneurship.

### A Leader’s and Entrepreneur’s Emergence as Well as Success Is Driven by Cognitive Ability

#### Leadership and Cognitive Ability

Leaders with higher cognitive ability are more successful at their job. Meta-analyses confirm this positive relation and further show that the link between cognitive ability and job success is weaker for leaders compared to non-leaders (Judge et al., 2004; Hoffman et al., 2011). In fact, in the long term, the leaders’ success^1^ seems to depend more strongly on their personality traits than on their cognitive ability (Hunter et al., 2006; Reichard et al., 2011).

However, there are certain situations and circumstances where cognitive ability is of particular importance – for example, when leaders work for a private compared to governmental organization (Hoffman et al., 2011) or they feel less rather than more stressed in their environment (Judge et al., 2004). Additionally, cognitive ability more strongly relates to leadership success when success is measured by objective criteria, such as quantified team performance, compared to subjective criteria like effectiveness ratings (Judge et al., 2004). In summary, cognitive ability positively relates to leadership success but this relation is weaker than for non-leading jobs and further varies among different situations and criteria.

#### Entrepreneurship and Cognitive Ability

The relevance of cognitive ability for entrepreneurship seems underexplored (Baum et al., 2011). In fact, no meta-analysis on entrepreneurial success has taken cognitive ability into account, and those primary studies that have, rarely used explicit ability measures but rather ability proxies, such as self-ratings on how often cognitive tasks are fulfilled (Demirel, 2012). Our investigation uses a well-known intelligence test for explicitly assessing cognitive ability and further examines its direct link to diverse criteria of entrepreneurial success^1^.

Existing research indicates that cognitive ability might be relevant for entrepreneurship. On the one hand, findings show that selected skills, which are related to cognitive ability, influence entrepreneurial status and performance. For instance, the ventures of entrepreneurs with higher practical intelligence show better annual growth rates (Baum et al., 2011) and entrepreneurs with higher divergent-thinking skills report more success and venture creation (Ames and Runco, 2005). On the other hand, cognitive ability might be vital for entrepreneurs in dealing with the relatively high complexity of their job (Busenitz and Barney, 1997). In that regard, Hartog et al. (2010) state that among different cognitive abilities, mathematical and technical ones are particularly valuable for entrepreneurs. Additionally, Sternberg (2004) argues that a combination of analytical, creative and practical intelligence is predominantly important in entrepreneurship. Finally, Roberts et al. (2007) conclude in their review that general mental ability is important for any occupational outcomes while Judge et al. (1999) highlight that cognitive ability predicts occupational status even across the life span. Based on these findings we assume cognitive ability to directly relate to entrepreneurial outcomes.

### A Leader’s and Entrepreneur’s Emergence as Well as Success Is Driven by Personality Traits

Most of the research focusing on the importance of personality traits has used the Five Factor Model of personality. It is undoubtedly the most common taxonomy to structure personality and comprises the five relatively stable traits Openness, Conscientiousness, Extraversion, Agreeableness and Emotional Stability (also known as Big Five; Costa and McCrae, 1992). To compare our findings to previous ones, we will also refer to the Big Five traits and subsequently summarize findings on their relevance for success in leadership and entrepreneurship.

#### Leadership and Big Five Traits

Leadership success clearly relates to the Big Five traits. Judge et al. (2004) demonstrated that the Big Five traits conjointly explain 23% of the variance in leadership performance. Their finding that not all traits are equally important is supported by other meta-analyses (e.g., Barrick et al., 2001). Extraversion relates strongest to the diverse criteria of leadership success, followed by conscientiousness, emotional stability and openness. Only agreeableness displays a relatively weak link, indicating that modesty, tact or sensitivity is not of high importance for a leader’s success. Even though these findings are relatively stable across cultures (Silverthorne, 2001), there are certain situations where the Big Five traits more strongly influence leadership success than in others. In essence, the link between the Big Five traits and success is most consistently moderated by the leader’s autonomy level, industry sector and success criterion. Personality traits like the Big Five are particularly important for a leader’s success when the leader operates in a highly autonomous work setting with a subjectively low stress level (Ng et al., 2008) and in private rather than governmental organizations (Hoffman et al., 2011). Moreover, personality traits more strongly relate to success when it is subjectively evaluated (e.g., performance ratings) compared to objectively measured (Hoffman et al., 2011). In summary, the Big Five traits relate to leadership success, but this relation differs across the five traits and is moderated by various situational and methodical aspects.

#### Entrepreneurship and Big Five Traits

The importance of the Big Five traits is clearly less examined in the context of entrepreneurship than leadership. The only two relevant meta-analyses show that entrepreneurs are more conscientious, emotionally stable and open but less agreeable than managers (Zhao and Seibert, 2006) and that the Big Five conjointly explain 37% of a person’s entrepreneurial status (Zhao et al., 2010). Moreover, they also relate to entrepreneurial success and venture growth, whereby conscientiousness and openness are, therefore, of particular importance (Zhao and Seibert, 2006).

Recent primary studies are largely in line with these meta-analytic findings and further demonstrate that the Big Five traits relate to a wider range of entrepreneurial success criteria, including sales and profitability growth or return on equity (Hachana et al., 2018). In this investigation, we build upon the reported findings and assume a direct link between the Big Five traits and entrepreneurial status as well as performance.

### This Investigation

This investigation examines whether socio-emotional skills add incremental validity over and interact with cognitive ability when predicting leadership and entrepreneurship (1) intention, (2) emergence, and (3) success. To this end, the socio-emotional skills we focus on are vocational interests and personality traits. To pursue the goals of this investigation, we present three studies which are tied together on the grounds of the Leader–Trait–Emergence–Effectiveness model (LTEE; Judge et al., 2009).

In line with the LTEE model, we see leadership and entrepreneurship intention as the first step toward a career in the field of leadership and entrepreneurship. Thus, the first study focuses on leadership and entrepreneurship intention and examines whether vocational interests add incremental value over and interact with cognitive ability when predicting it. Leadership and entrepreneurship intention enhance the chance of achieving a leadership or entrepreneurship position which, in turn, is a necessity for achieving leadership and entrepreneurship success (Judge et al., 2009). In line with this sequential connection between leadership and entrepreneurship intention, emergence and success, the two remaining studies focus on leadership and entrepreneurship emergence as well as success. Here it is worth mentioning that Study 2 and 3 focus on the incremental validity of personality traits rather than vocational interests because personality traits seem more forceful socio-emotional skills for leadership and entrepreneurship emergence and success than vocational interests. In brief, Study 2 examines the incremental validity of the Big Five personality traits over cognitive ability when predicting leadership emergence and success while Study 3 investigates the incremental validity and interaction with regard to entrepreneurship status and success. For the sake of clarity, Figure 1 summarizes the key aspects of all three studies.

With respect to Study 1 we derive our hypotheses in accordance to the LTEE model. Thus, we assume that cognitive ability enhances a person’s leadership and entrepreneurship intention because jobs in leadership and entrepreneurship offer cognitive complexity, which should particularly attract persons with higher ability levels. At the same time the person–environment fit theory (Kristof, 1996) suggests that individuals search for careers that match their vocational interests (Holland, 1959). As we assume a certain fit for leadership and entrepreneurship careers with enterprising, conventional and social interests, we assume that persons with these interests express higher leadership and entrepreneurship intention. Moreover, because becoming a leader or entrepreneur naturally comes with problems that challenge the person’s cognitive ability as well as their career aspiration, both cognitive ability and vocational interests are simultaneously needed to sustain the respective career intention. Thus, we assume that a person’s cognitive ability predicts his/her leadership and entrepreneurship intention while this prediction is enhanced by simultaneously considering this person’s enterprising, social and conventional interests. In a more explorative manner we further assume that these vocational interest interact with cognitive ability when predicting leadership and entrepreneurship intention as vocational interest show reciprocal influence in the career choice process (Ackerman and Beier, 2003). Thus, the following hypothesis (H) and explorative research question (RQ) are stated:

**H1: The vocational interests enterprising, social and conventional conjointly add incremental value beyond cognitive ability when predicting a) leadership intention and b) entrepreneurial intention.**

**RQ1: The vocational interests enterprising, social and conventional moderate the link between cognitive ability and a) leadership intention and b) entrepreneurial intention.**

Leadership and entrepreneurship intention enhance the chance of emerging as a leader or entrepreneur and achieving success as such (Chan and Drasgow, 2001; Judge et al., 2009). The prediction of leadership and entrepreneurship emergence as well as success is examined in Study 2 and 3. In line with the LTEE model, we argue that cognitive ability and the Big Five traits are *both* predictors of success and as such are *both* important for reaching and effectively fulfilling jobs in leadership and entrepreneurship. As previously summarized, empirical evidence supports this assumption for leadership and entrepreneurship (e.g., Judge et al., 2004; Zhao and Seibert, 2006).

Importantly, on the grounds of the person–environment fit theory (Kristof, 1996), we further claim that the Big Five traits are particularly important when leading or founding a business as both require interpersonal behavior, which is strongly influenced by them. In fact, success in leadership and entrepreneurship jobs may rely even more strongly on interpersonal behavior than success in other jobs as interpersonal interactions are part of everyday business in leadership and entrepreneurship (Burke et al., 2006; Elmuti et al., 2012). Because successfully managing interpersonal interactions is determined by a person’s Big Five traits rather than by his/her cognitive ability (Ackerman and Heggestad, 1997), we assume that the Big Five traits add valid information beyond cognitive ability when predicting leadership success and entrepreneurial success. Even though the incremental validity of the Big Five traits over cognitive ability has been proven for success in a variety of contexts (e.g., school performance; Bratko et al., 2006), only little is known in the leadership context and even less is recognized in the entrepreneurship context. However, as leadership jobs resemble entrepreneurial ones (Czarniawska-Joerges and Wolff, 1991), we assume for both job-types that the Big Five traits add valid information beyond cognitive ability when forecasting success. Some studies even suggest that cognitive ability moderates the link between the Big Five traits and occupational outcomes (see for an overview Mount et al., 1999) for why it is also explored whether the Big Five traits interact with cognitive ability when predicting the emergence of top-level leadership and entrepreneurship as well as leadership and entrepreneurship success. Thus, the following hypotheses and research questions are stated:

**H2: The Big Five personality traits conjointly add incremental value beyond cognitive ability when predicting a) top-level leader emergence and the success criteria b) income and c) supervisor-rated leadership performance.**

**RQ2: The Big Five personality traits moderate the link between cognitive ability and a) top-level leader emergence and the success criteria b) income and c) supervisor-rated leadership performance.**

**H3: The Big Five personality traits conjointly add incremental value beyond cognitive ability when predicting a) entrepreneurial status and b) the success criterion self-rated entrepreneurial performance.**

**RQ3: The Big Five personality traits moderate the link between cognitive ability and a) entrepreneurial status as well as b) self-rated entrepreneurial performance.**

## Study 1: Leadership and Entrepreneurship Intention and their Link to Cognitive Ability and Interests

### Method of Study 1

#### Sample and Data Collection

In Study 1, a total of 420 Austrian students (61% female, 39% male) took part and were assessed twice online (T1, T2). A certain drop had to be dealt with due to incomplete datasets for the last wave of this long-term study. Altogether, 231 students (58% female, 42% male) provided complete data for both timepoints and were subsequently used to examine the hypotheses. These participants were on average 17 years old (*SD* = 4.14), had one sibling (*SD* = 0.84) and 40% of them had parents who were self- employed.

All participants were contacted during an informative event on career choices. These events are nationwide initiatives for students at the end of their scholastic education organized by the Federal Ministry of Education to inform them about potential jobs and job training. The events are free of charge, participation is voluntary, and students are usually encouraged by their schools to attend them. For study participation, students were offered two vouchers for an online retailer (one at each testing time). Participants provided data on their cognitive ability and vocational interests at T1 and agreed to be contacted again. Two years later (T2), participants were again contacted and completed questions on their leadership intention and entrepreneurship intention.

#### Measures

**Predictors: Cognitive ability and vocational interests**

##### Cognitive ability

The ability measure administered was the German Intelligence Structure Test 2000-R (IST-R; Amthauer et al., 2001), which includes items on verbal, numerical and figural intelligence in a basic module. All correctly answered items were summed up to form the cognitive ability score. This score was then converted into IQ-values with a mean of 100 and a standard deviation of 15. The cognitive ability score is thought to measure reasoning as a higher order factor of intelligence and is also referred to as general mental ability (GMA).

##### Vocational interests

Interests were assessed with the General Interest Structure Test (German version; Bergmann and Eder, 2005), which measures Holland’s (1959) interest model. Thirty items represented activities that matched either enterprising, social or conventional interests. All items were completed on a five-point scale ranging from 1 (*I am not interested in this at all; I do not enjoy doing this at all*) to 5 (*I am very interested in this; I enjoy doing this very much)*. Internal consistencies are presented in Table 1. In this study, vocational interests represent socio-emotional skill which are thought to influence leadership and entrepreneurship intention beyond cognitive ability.

**TABLE 1:** Means (M), standard deviations (SD), Cronbach’s α (diagonal), and intercorrelations among variables (Study 1).

	*M*	*SD*	Age	Gender	Back- ground	Leader. Intent.	Entrep. Intent.	Cogn. Ability	Enter- prising	Social	Conven-tional
Age	16.60	4.14	−								
Gender	−	−	−0.20**	−							
Family Background	−	−	0.02	0.02	−						
Leadership Intent.	3.41	1.25	–0.08	0.18**	0.09	−					
Entrepreneurship Intent.	2.84	1.39	–0.02	0.19**	0.10	0.54**	−				
Cognitive Ability	98.74	14.93	0.34**	0.01	–0.01	0.14*	0.14*	−			
Enterprising	3.18	0.83	0.11^†^	–0.05	0.10	0.37**	0.29**	0.08	0.83		
Social	3.23	0.86	0.13*	−0.51**	–0.01	–0.03	0.01	0.02	0.38**	0.75	
Conventional	2.68	0.72	0.30**	0.10	0.08	0.14*	0.08	0.24**	0.36**	−0.03**	0.85

*n* = 231; **p ≤ 0.01, *p ≤ 0.05, ^†^*p* ≤ 0.10.

##### Control variables

Gender (dummy-coded; 0 = female, 1 = male) and age were control variables as they relate to vocational interests and leadership as well as entrepreneurship intention (e.g., Hirschi and Läge, 2007; Lippa, 2010; Lechner et al., 2018). Additionally, we controlled for parental role modeling as this too correlates with vocational interests and the intention for leadership or entrepreneurial roles (e.g., Palmer et al., 2019). Parental role modeling was measured by whether at least one parent was self-employed or an entrepreneur at the beginning of the study (0 = no entrepreneur among parents, 1 = entrepreneur among parents).

##### Criteria: leadership and entrepreneurship intention

###### Future leadership intention

A German translation of Singer’s (1991) single-item scale “I am motivated to take over a leadership position at work” was completed on a five-point scale ranging from 1 (*strongly disagree*) to 5 (*strongly agree*) to assess leadership intention 2 years after measuring cognitive ability and the Big Five traits.

###### Future entrepreneurship intention

Entrepreneurial intention was measured by a single-item scale adapted from Liñán and Chen (2009): “I have the firm intention to start a firm some day.” Responses were given on a five-point scale ranging from 1 (*very unlikely*) to 5 (*very likely*) 2 years after measuring cognitive ability and the Big Five traits.

### Results of Study 1

Table 1 shows the descriptive statistics, reliabilities and correlations for all variables in Study 1. Internal consistencies range from 0.75 to 0.85 and show good reliability for the respective measurements. General mental ability (GMA) correlates with leadership intention and entrepreneurship intention (both *r* = 0.14, *p* ≤ 0.05). Additionally, enterprising and conventional interests relate to leadership intention (*r*_enterp._ = 0.37, *p* ≤ 01; *r*_conv._ = 0.14, *p* ≤ 05), while enterprising is also linked to entrepreneurship intention (*r* = 0.29, *p* ≤ 01).

To test hypothesis 1, we studied the incremental validity of vocational interests over general mental ability when predicting leadership intention and entrepreneurship intention. A stepwise hierarchical regression was used and GMA was entered first, followed by the interests enterprising, social and conventional in a next step. To consider age-related, gender-related and background-driven differences in interests and leadership or entrepreneurship intention, we controlled for study participants’ age, gender and entrepreneurial family background. Regression results are summarized in Table 2 and confirm hypotheses 1a and 1b. Thus, vocational interests add incremental validity over GMA when predicting future leadership intention (H1a: Δ*R*^2^ = 15%, *p* ≤ 0.01) and future entrepreneurship intention (H1b: Δ*R*^2^ = 9%, *p* ≤ 0.01).

**TABLE 2:** Hierarchical regression predicting leadership and entrepreneurship intention from control variables, cognitive ability, and vocational interests (Study 1).

		Leadership intention			Entrepreneurship intention		
		Linear regression			Linear regression		
Block	Predictors	*R*^2^_*adj.*_	Δ*R*^2^	β	*R*^2^*_adj._*	Δ*R^2^*	β
1	Control Variables	0.03*	0.04*		0.03*	0.05*	

2	Block_1_ + Ability	0.05**	0.03**		0.05**	0.02*	
	Cognitive Ability			0.15*			0.15*

3	Block_2_ + Interests	0.20**	0.15**		0.12**	0.09**	
	Enterprising			0.45**			0.34**
	Social			−0.15*			–0.05
	Conventional			–0.07			–0.10

4	Block_3_ + Interaction	0.21**	0.03*		0.12**	0.00	
	Ability*Enterprising			–0.07			–0.04
	Ability*Social			0.16*			0.07
	Ability*Conventional			–0.05			–0.01

*n* = 231; **p ≤ 0.01, *p ≤ 0.05, ^†^*p* ≤ 0.10. Control variables include age, gender and entrepreneurial family background.

The direction of the effects and the relative importance of the three vocational interests is represented by the β-values in Table 2. Results show that higher enterprising interests predict a person’s intention to take on future leadership roles best (β = 0.45, *p* ≤ 0.01). Additionally, the higher a person’s social interests, the lower is his/her intention to take on leadership positions in the future (β = −0.15, *p* ≤ 0.05). With respect to entrepreneurship intention, similar results were found. Higher enterprising interests predict a person’s future entrepreneurship intention best (β = 0.34, *p* ≤ 0.01). With respect to these regression results, it is worth mentioning that after considering the control variables, GMA only predicted 5% of the variance in future leadership intention, while GMA and the three vocational interests conjointly explained 20%. Regarding the entrepreneurship intention, GMA predicted 5% of its variance, while GMA and the three vocational interests conjointly explained a total of 12%.

**FIGURE 2:**
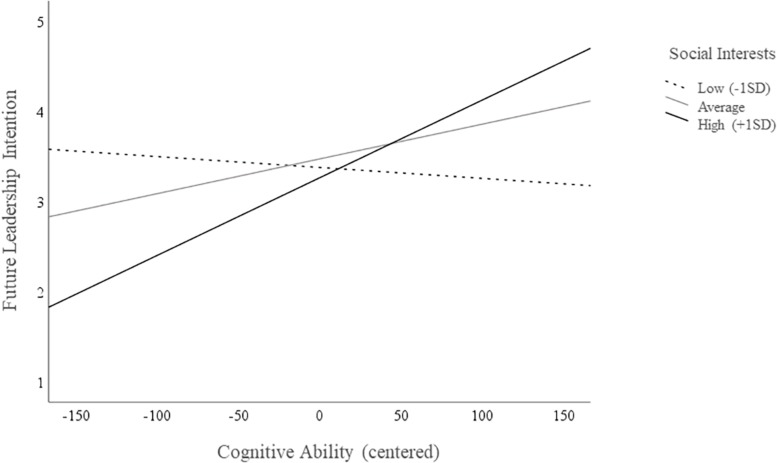
Relationship of cognitive ability to leadership intention for three levels of social interest.

To test the research question 1, we examined whether vocational interests and cognitive ability interact when predicting leadership and entrepreneurship intention. Interaction effects were studied using regression analyses which are summarized in Table 2. All predictors were centered around their means (Aiken and West, 1991) before computing the interaction terms and entering the variables into the regression model. The results indicate a significant interaction only for social interests and cognitive ability when predicting future leadership intention (β = 0.16, *p* ≤ 0.05, Δ*R*^2^ = 3%, *p* ≤ 0.05). To probe the interaction, simple effect coefficients were computed using three levels of social interests, one *SD* below the mean, at the mean and one *SD* above the mean. Figure 2 graphs the interaction, showing the change in leadership intention through cognitive ability at the different levels of social interest. The interaction suggests that when individuals report high social interests then their intention to take on a leader role is more strongly influenced by cognitive ability than when they express low social interests. In fact, those with the highest leadership intention show both, high social interests and high cognitive ability. Thus, research question 1 gets support for social interests.

### Brief Discussion of Study 1

Study 1 demonstrates that those with higher cognitive ability are drawn to careers in both leadership and entrepreneurship. Importantly, vocational interests improve the cognitive-based prediction of who intends to become a business leader or entrepreneur by up to 15 percent. With regard to the former, individuals with higher enterprising interest report higher intention to take on a leadership role in the future while those with higher social interests show less intention for such a role. Interestingly and anew, the interaction results show that social interests enhance the leadership intention of those with higher cognitive ability while they reduce the intention of those with lower cognitive ability. While social interests only predict leadership intention, enterprising interests relate to both, leadership and entrepreneurship intention. Individuals with higher enterprising interests also report higher intention to found their own business.

With regard to the importance of enterprising interests, it might be argued that their effect on leadership and entrepreneurship intention is based on certain personality traits that are linked to both enterprising and leadership or entrepreneurship intention. This assumption builds on findings showing a positive link between enterprising interests and traits such as self-efficacy, extraversion, achievement-orientation and personal initiative (Chan and Drasgow, 2001; Bergmann and Eder, 2005). As these traits, for instance, also relate to leadership intention (Stiehl et al., 2015), the observed effect might be due to the circumstance that people with stronger enterprising interests are also more extraverted and achievement-oriented, which in turn goes hand in hand with higher intentions to lead and found a business.

In brief, it can be summarized that the future intention to take on leadership or entrepreneurship roles is only marginally based on a person’s cognitive ability, yet it is clearly influenced by vocational interests and also partly by the interaction of these with cognitive ability. Finally, it is worth mentioning that this study set its focus on leadership and entrepreneurship intention as a prerequisite of actually emerging and successfully acting as a leader or entrepreneur while the following studies will address the emergence and success of leaders as well as entrepreneurs.

## Study 2: Leadership Level and Success and their Link to Cognitive Ability and the Big Five Traits

### Method of Study 2

#### Sample and Data Collection

In Study 2, a total of 142 leaders from Austria (49% female, 51% male) took part. Participation was possible during a leadership development program. Once the participation started, intermitting it was possible in case the leaders had to fully concentrate on the development program. This possibility for intermission led to the circumstance that a selected number of leaders did not fully complete their study participation but lacked data (mainly with respect to the ability score) for why they were excluded from the sample. Overall, 123 leaders (50% ♀) with a mean age of 40 years (*SD* = 7.68) provided complete information on their cognitive ability, Big Five traits and the three criteria (1) leadership level, (2) income, and (3) supervisor-rated leadership performance.

The leaders came from the service industry (logistics, delivery services, parcel delivery), had served, on average, for 5 years (*SD* = 26.91) in their current employment, and were responsible for 1 to 300 subordinates (*Mdn* = 5, *M* = 14.90, *SD* = 32.91). Participation was voluntary, anonymous and for research purposes only. The participants were contacted during a company-wide leadership development program. Those who chose to take part in the survey completed an online version of the subsequent measures. Furthermore, participants named their direct supervisor who was then contacted to evaluated the participant’s leadership performance. All questionnaires were provided in German as this was the participants’ first language. Participants received written feedback on their personality scores to compensate them for their efforts.

#### Measures

**Predictors: Cognitive ability and personality traits**

##### Cognitive ability

The Wonderlic Personnel Test (German version; Wonderlic and Associates, 1992) was used to assess general mental ability (GMA). This 50-item test is administered in 12 min and the ability score is calculated by summing the number of correct answers given in the allotted time. This score is then converted into IQ-values with a mean of 100 and a standard deviation of 15.

##### Personality traits

The Big Five traits were assessed using the NEO-Five-Factor Inventory (Costa and McCrae, 1992; German version: Borkenau and Ostendorf, 1993) which comprises 60 items that are rated on a five-point Likert scale ranging from 1 (*strongly disagree*) to 5 (*strongly agree*). Each of the five personality traits is assessed by 12 items. Internal consistencies are presented in Table 3. In this study, the Big Five traits represent socio-emotional skill which are thought to influence leadership level and success beyond cognitive ability.

**TABLE 3:** Means (M), standard deviations (SD), Cronbach’s α (diagonal), and intercorrelations among variables (Study 2).

	*M*	*SD*	Gender	Age	Years in position	Income	Leadership level	Perfor-mance	Cogn. Ability	O	C	E	A	Est
Gender	−	−	−											
Age	39.49	7.68	−0.17^†^	−										
Years in position	44.90	45.90	–0.09	0.41**	−									
Income	4.12	1.08	−0.31**	0.17^†^	–0.01	−								
Leadership Level	2.85	1.45	−0.26**	0.30**	–0.06	0.43**	−							
Performance	3.71	0.59	–0.11	–0.11	–0.04	0.15^†^	0.14	0.84						
Cognitive Ability	100.02	15.00	−0.18*	−0.26**	−0.20*	0.22*	0.14^†^	0.24**	−					
Openness (O)	2.77	0.46	0.25**	–0.10	–0.05	−0.16^†^	–0.11	0.03	0.08	0.75				
Conscientiousness (C)	3.16	0.43	0.20*	−0.24**	0.32	0.03	–0.11	0.24**	–0.03	0.15^†^	0.79			
Extraversion (E)	2.86	0.43	0.09	–0.03	0.05	0.19*	0.07	0.28**	–0.08	0.24**	0.32**	0.70		
Agreeableness (A)	2.84	0.38	–0.02	–0.02	–0.07	0.04	–0.05	0.00	0.01	0.30**	0.04	0.13	0.68	
Emo. St-bility (Est)	3.73	0.55	0.06	–0.06	0.09	0.08	−0.22*	0.29**	–0.04	0.09	0.37**	0.38**	0.19*	0.85

*n* = 123 leaders. Gender is dummy-coded (0 = male, 1 = female). **p ≤ 0.01, *p ≤ 0.05, ^†^*p* ≤ 0.10.

##### Control variables

Gender (dummy-coded: 0 = male, 1 = female), age and number of years in the current employment were used as control variables because they are known to relate to both personality traits and diverse leadership success criteria (e.g., Weichselbaumer and Winter-Ebmer, 2005; Weisberg et al., 2011).

##### Criteria: leadership level and success

###### Leadership level

The higher a leader’s position in the hierarchy, the higher is this person’s leadership level. In accordance with Tharenou et al. (1994), leaders were asked to provide their level in the organization’s hierarchy. They had to choose from the following six categories, which adequately represented the organizations’ management structure: 1 (*project leader*); 2 (*team leader*); 3 (*department leader*); 4 (*division leader*); 5 (*branch leader*); 6 (*board member, (vice-) president or CEO*).

###### Income as a criterion for objective leadership success

The amount of a leader’s income is commonly used to measure this leader’s success in a rather objective way. In line with Judge et al. (1999), the participants of this study were asked to rate their yearly income (after tax). Six categories ranging from “less than 12,000 euros” to “more than 66,000 euros” were used and subsequently coded from 1 to 6, with higher scores reflecting higher income.

##### Supervisor-rated leadership performance as a criterion for subjective success

In accordance with research measuring leadership success with both objective and subjective success criteria, this study uses performance ratings in addition to the objective criteria. As is common in leadership research, we used ratings of the target leader’s direct supervisor. Each supervisor provided ratings on the target leader’s task and contextual performance. Following Scullen et al. (2003), leadership task performance refers to task-specific behaviors, including technical and administrative core responsibilities of leaders (e.g., accounting, planning, organizing work). Leadership contextual refers to a leader’s interpersonal performance, particularly motivational behavior and maintaining interpersonal relationships (e.g., supporting, cooperating). Task and contextual performance were measured with four items each, adapted from Bergner et al. (2010). The items were rated on a five-point Likert scale ranging from 1 (*strongly disagree*) to 5 (*strongly agree*) and were averaged to form a composite score on supervisor-rated leadership performance. Table 3 shows the internal validity of the composite score.

### Results of Study 2

Table 3 displays the descriptive statistics, reliabilities and correlations for all variables in Study 2. Cronbach’s α values range from 0.66 to 0.79 and show acceptable-to-good reliability for the respective measurements. GMA correlates with the leadership success criteria income (*r* = 0.22, *p* ≤ 0.05), leadership level (*r* = 0.14, *p* ≤ 0.10) and supervisor-rated leadership performance (*r* = 0.24, *p* ≤ 0.01). Among the Big Five traits, openness, extraversion and emotional stability are related to at least one leadership success criterion.

To test hypothesis 2 and thus examine the incremental validity of the Big Five traits over cognitive ability when predicting top-level leader emergence and success, stepwise hierarchical regressions were used. Table 4 summarizes the regression results and also shows that analyses are controlled for age-related, gender-related and experience-related effects. With respect to the Big Five’s incremental value, the results support hypotheses 2a, 2b and 2c. The Big Five traits add validity beyond GMA when predicting leadership level (H2a: Δ*R*^2^ = 8%, *p* ≤ 0.05), income (H2b: Δ*R*^2^ = 9%, *p* ≤ 0.05) and supervisor-rated leadership performance (H2c: Δ*R*^2^ = 15%, *p* ≤ 0.05). The β-values in Table 4 show that emotional stability negatively and extraversion positively correlate with leadership level (β_*stability*_ = −0.23, *p* ≤ 0.01; β_*extraversion*_ = 0.22, *p* ≤ 0.05) while extraversion positively and openness negatively relate to income (β_*extraversion*_ = 0.27, *p* ≤ 0.01; β_*openness*_ = −0.21, *p* ≤ 0.05). Finally, emotional stability and extraversion both positively relate to supervisor-rated leadership performance (β_*stability*_ = 0.22, *p* ≤ 0.01; β_*extraversion*_ = 0.24, *p* ≤ 0.01). When considering the control variables, Big Five and GMA conjointly, then they explain 22% of the variance in leadership level, 19% of the variance in income and 16% of the variance in supervisor-rated leadership performance.

**TABLE 4:** Hierarchical regression predicting leaders’ income, leadership level, and supervisor-rated leadership performance from control variables, cognitive ability, and the Big Five traits (Study 2).

		Leadership level			Income			Supervisor-rated leadership performance		
		Linear regression			Linear regression			Linear regression		
Block	Predictors	*R*^2^*_adj._*	Δ*R^2^*	β	*R*^2^*_adj._*	Δ*R^2^*	β	*R*^2^*_adj._*	Δ*R^2^*	β
1	CV	0.15**	0.17**		0.09**	0.11**		0.00	0.03	

2	Block_1_ + Ability	0.17**	0.03^†^		0.13**	0.04*		0.04^†^	0.04*	
	Cognitive Ability			0.20*			0.25**			0.23*

3	Block_2_ + Big Five	0.22**	0.08*		0.19**	0.09*		0.16**	0.15**	
	Openness			–0.10			−0.21*			–0.04
	Conscientiousness			0.09			0.07			0.11
	Extraversion			0.22*			0.27**			0.24*
	Agreeableness			–0.01			0.05			–0.07
	Emo. Stability			−0.23**		–0.04	–0.04			0.22*

4	Block_3_ + Interaction	0.23**	0.04		0.23**	0.07^†^		0.16**	0.02	
	Openness*Ability			0.13			0.23*			0.13
	Conscientious.*Ability		–0.11			0.08			0.05	
	Extraversion*Ability			0.11			0.06			0.05
	Agreeableness*Ability			0.06			–0.08			0.02
	Emo. Stability*Ability			−0.19*		−0.24*				0.10

*n* = 123 leaders. CV = Control variables (age in years, gender dummy-coded, years in current position). **p ≤ 0.01, *p ≤ 0.05, ^†^*p* ≤ 0.10.

**FIGURE 3:**
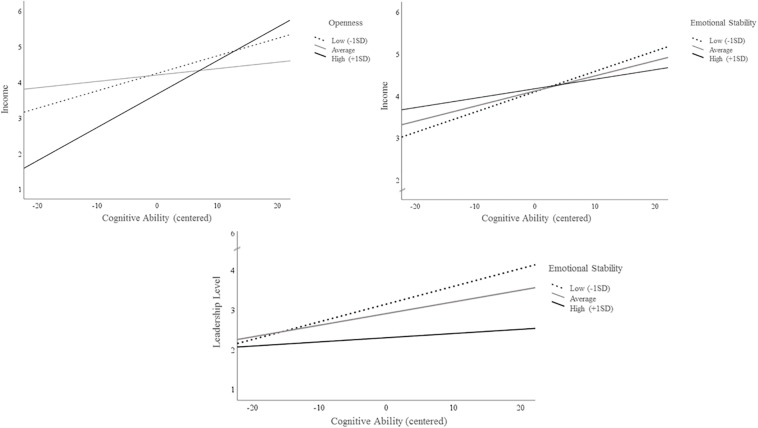
Relationship of cognitive ability to leadership level and success for three levels of emotional stability and openness.

To test the research question 2, it was studied whether the Big Five traits interact with cognitive ability when predicting leadership level, income and supervisor-rated leadership performance. Interaction effects were examined using regression analyses which are summarized in Table 4. All predictors were centered around their means (Aiken and West, 1991) for the analyses. The results show significant interactions when predicting leadership level (Emotional Stability^∗^Ability: β = −0.19, *p* ≤ 0.05, Δ*R*^2^ = 4%, *p* > 0.05) and income (Emotional Stability^∗^Ability: β = −0.24, *p* ≤ 0.05 and Openness^∗^Ability: β = 0.23, *p* ≤ 0.05, Δ*R*^2^ = 7%, *p* ≤ *0.10*). Figure 3 graphs the interactions and shows that when leaders are emotionally instable their leadership level and income more strongly depend on their cognitive ability. The same holds true for a leader’s income when he/she shows high openness to new experiences. Thus, research question 2 gets partial support.

### Brief Discussion of Study 2

Study 2 shows that the Big Five traits enhance the ability-driven prediction of a leader’s hierarchical level, income and performance. While extraversion is of importance for all these success criteria, openness and emotional stability improve the prediction only of selected criteria. The particular importance of extraversion is in line with previous studies, which show that on the long run, the leaders’ success rather depends on their extraversion than on their cognitive ability (Reichard et al., 2011). Moreover, the current findings support the importance of personality traits beyond cognitive ability in a wider context, as they confirm results from South Korea where personality traits were seen to add moderate incremental validity beyond cognitive ability for the contextual performance of military leaders (Oh et al., 2014).

Importantly and anew, emotional stability and openness interact with cognitive ability when predicting selected leadership outcomes. Openness seems to facilitate the impact of cognitive ability on a leader’s income: highly open-minded leaders profit more from their cognitive ability when it comes to their income compared to less open-minded ones. In contrast, emotional stability seems to buffer the effect of cognitive ability on a leader’s income so that emotionally stable leaders with higher ability report lower income than their more neurotic but equally clever colleagues. Emotional stability also marginally interacts with cognitive ability when predicting a leader’s hierarchical level. Interestingly, leaders with similar cognitive ability end up in higher positions when they are more neurotic.

In conclusion, being smart seems not enough for achieving high leadership positions and success as a leader. Importantly, the Big Five traits enhance a leader’s success irrespective of his/her cognitive ability and some traits even buffer or enhance the effect of cognitive ability. The subsequent Study 3 extends these findings to the field of entrepreneurship.

## Study 3: Entrepreneurship Status and Success and their Link to Cognitive Ability and the Big Five Traits

### Method of Study 3

#### Sample and Data Collection

In Study 3, a total of 162 Austrian participants (38% female, 62% male) from various working fields including business, law, technology, arts and social science took part. Of those, seven had to be excluded due to incomplete ability data (they completed less than 10 ability items). The remaining 155 participants (35% female, 65% male) were on average 29 years old (*SD* = 6.84) and varied regarding their educational background. Overall, 60% held a university degree and 34% an A-level, 6% served an apprenticeship or vocational training. Out of these 155 participants, 47% had already founded a business, which they currently managed, which is why they are referred to as entrepreneurs. In contrast, 53% were employed and had neither founded nor run a business on their own, which is why they are termed non-entrepreneurs in this study. Contrary to and more beneficial than in other studies, our non-entrepreneurial sample does not encompass university students but includes individuals who are employed. The entrepreneurs and non-entrepreneurs were matched regarding gender; thus, men and women were equally distributed across the groups. However, entrepreneurs were older than non-entrepreneurs (*M*_*E*_ = 31.89, *SD*_*E*_ = 6.99 vs. *M*_*non–E*_ = 26.13, *SD*_*non–E*_ = 5.43, *t*_153_ = 5.74, *p* ≤ 0.01).

Data were collected in Austria through an online survey, which was sent out to participants of business talks, trade fairs and vocational networking events. All these events were organized by the chamber of commerce and targeted individuals who aim to extend their vocational network. Participation in these events and in our study was voluntary. Participants provided information on their cognitive ability, Big Five traits and their entrepreneurial status (yes/no) by completing a German version of the subsequent measures. Those who were entrepreneurs further rated their entrepreneurial performance within the last 12 months. All received written feedback on their personality scores to compensate them for their efforts and consented to the use of their data for research purposes; they were guaranteed that no personalized data would be passed on.

#### Measures

**Predictors: Cognitive ability and personality traits**

##### Cognitive ability

The Wonderlic Personnel Test (German version; Wonderlic and Associates, 1992) was used to assess general mental ability (GMA). Its 50 items refer to verbal, numerical and figural tasks. The ability score is calculated by summing the number of correct answers given within 12 min. This score is converted into IQ-values with a mean of 100 and a standard deviation of 15.

##### Personality traits

The Big Five traits were assessed using the Big Five Inventory (Rammstedt and John, 2005), which consists of 21 German items that are completed on a five-point Likert scale ranging from 1 (*strongly disagree*) to 5 (*strongly agree*). Scores for all trait dimensions show satisfactory-to-good internal consistencies (see Table 5). Here, the Big Five traits represent socio-emotional skill which are thought to influence entrepreneurship status and success beyond cognitive ability.

**TABLE 5:** Means (M), standard deviations (SD), Cronbach’s α (diagonal), and intercorrelations among variables (Study 3).

	*M*	*SD*	Age	Gender	Edu-cation	Entre. Perform.	Entre. Status	Cogn. Ability	O	C	E	A	Est
Age	28.81	6.84	−										
Gender	−	−	−0.23**	−									
Educational Background	1.45	0.63	0.02	0.13^†^	−								
Entrepreneurial Perform.	3.89	0.93	0.03	0.10	0.22^†^	0.89							
Entrepreneurial Status	−	−	0.42**	–0.03	–0.04	−	−						
Cognitive Ability	100.52	14.23	0.02	0.11	0.01	–0.01	0.17*	−					
Openness (O)	3.85	0.61	0.16*	–0.09	0.06	–0.02	0.26**	–0.03	0.60				
Conscientiousness (C)	3.87	0.61	0.16*	0.06	–0.01	0.32**	0.13^†^	–0.03	0.08	0.67			
Extraversion (E)	3.58	0.79	–0.05	–0.08	–0.02	−0.20^†^	0.04	0.07	0.10	0.09	0.82		
Agreeableness (A)	3.08	0.75	0.04	–0.09	–0.03	–0.14	–0.05	0.06	0.07	0.04	0.31**	0.70	
Emo. Stability (Est)	3.66	0.79	0.12	0.21**	–0.04	−0.23*	0.16*	0.13	0.02	0.15*	0.30**	0.20**	0.70

*n* = 162 except for entrepreneurial performance where *n* = 72 entrepreneurs. Entrepreneurial status is dichotomously coded (yes/no) and thus values represent point-biserial correlations. **p ≤ 0.01, *p ≤ 0.05, ^†^*p* ≤ 0.10.

##### Control variables

Gender (dummy-coded; 0 = female, 1 = male), age and educational background (1 = university, 2 = A-level, 3 = specific vocational training, 4 = apprenticeship, 5 = compulsory education) were control variables as they relate to both personality traits and cognitive ability as well as assorted criteria of entrepreneurship success (e.g., Weisberg et al., 2011; Thorgren et al., 2016).

##### Criteria: entrepreneurship status and success

###### Entrepreneurship status

Entrepreneurship status was defined as being active as an entrepreneur at the time of the investigation (yes = 1; no = 0). Our definition of entrepreneurs is based on the one used by Zhao and Seibert (2006) and considers somebody as an entrepreneur who is the founder, owner and manager of a business. Entrepreneurs were asked to provide their VAT-number to prove that they currently owned and managed their named business.

###### Entrepreneurship success

The fact that there is no commonly accepted measure for entrepreneurial success (Herman and Renz, 2004) led to the use of various measures like profit margin, employee turnover or job generation. Because business owners tend not to reveal their business financial data, and objective performance criteria are known to be contaminated (Naman and Slevin, 1993), research suggests also considering subjective performance ratings (Binder and Coad, 2013). We follow this suggestion and use self-report measures of entrepreneurial performance like previous research did (e.g., Axtell et al., 2006). Drawing on Jong et al. (2015), entrepreneurial performance was assessed with four items indicating the entrepreneur has improved current products/services, proactively acquired new customers, increased profit, and felt they had been performing well within the past 12 months. Items were completed on a five-point scale ranging from 1 (*strongly disagree*) to 5 (*strongly agree*) and finally averaged for calculating a composite score (see Table 5 for Cronbach Alpha value).

### Results

Table 5 shows the descriptive statistics, reliabilities and correlations for all variables in Study 3. Overall, entrepreneurs show higher scores on cognitive ability (*M*_*E*_ = 103.03, *SD*_*E*_ = 13.82 vs. *M*_*non–E*_ = 98.03, *SD*_*non–E*_ = 14.48, *t*_160_ = −2.26, *p* ≤ 0.05), emotional stability (*M*_*E*_ = 3.84, *SD*_*E*_ = 0.70 vs. *M*_*non–E*_ = 3.46, *SD*_*non–E*_ = 0.75, *t*_153_ = −2.50, *p* ≤ 0.05) and openness (*M*_*E*_ = 4.02, *SD*_*E*_ = 0.57 vs. *M*_*non–E*_ = 3.70, *SD*_*non–E*_ = 0.60, *t*_153_ = −3.35, *p* ≤ 0.01).

To test hypothesis 3, we studied the incremental validity of the Big Five traits beyond cognitive ability when predicting entrepreneurship status (yes/no) and self-perceived entrepreneurial performance. The analyses were controlled for participants’ age, gender and educational background. The hierarchical logistic regression in Table 6 shows that the Big Five traits add 7% over cognitive ability when predicting a person’s entrepreneurial status. Thus, H3a is confirmed. However, only openness was a significant predictor, indicating that more open individuals are also more likely to become entrepreneurs (*Exp(B)* = 2.62, *p* ≤ 0.01). The predicted probabilities further show that the entrepreneurship status (yes/no) of 71% was correctly classified when solely considering their cognitive ability and control variables. When the Big Five traits were added, the number of correctly classified participants significantly increased to 73%.

**TABLE 6:** Hierarchical regression predicting entrepreneurial status and performance from control variables, cognitive ability, and the Big Five traits (Study 3).

		Entrepreneurial status			Self-rated entrepreneurial performance		
		Log. regression (*n* = 162)			Linear regression (*n* = 72)		
Block	Predictors	*R*^2^_*Nag.*_	Δ*R^2^*	*Exp(B)*	*R*^2^*_adj._*	Δ*R^2^*	β
1	CV	0.25**	0.25**		0.01	0.05	0.00

2	Block_1_ + Ability	0.28**	0.03*		0.00	0.00	
	Cognitive Ability			1.10*			0.06

3	Block_2_ + Big Five	0.35**	0.07*		0.13*	0.18*	
	Openness			2.62**			0.05
	Conscientiousness			1.25			0.29*
	Extraversion			1.16			–0.13
	Agreeableness			0.70			–0.09
	Emo. Stability			1.16			0.24^†^

4	Block_3_ + Interaction	0.35**	0.08		0.14*	0.07	
	Openness*Ability			1.07			–0.05
	Conscientious*Ability			0.94			−0.28*
	Extraversion*Ability			0.96			–0.17
	Agreeableness*Ability			1.01			–0.01
	Emo. Stability*Ability			1.12*			0.00

**p ≤ 0.01, *p ≤ 0.05, ^†^*p* ≤ 0.10. CV = Control variables (age, gender dummy-coded, educational background).

In addition, the linear hierarchical regression in Table 6 revealed that the Big Five traits added incremental validity over cognitive ability when predicting self-rated entrepreneurial performance (H3b: Δ*R*^2^ = 18%, *p* ≤ 0.05). Thus, H3b was also supported. The more conscientious and emotionally stable somebody was, the more successful they perceived their performance as an entrepreneur (β*_*conscientious*_.* = 0.29, *p* ≤ 0.05; β*_*stability*_* = 0.24, *p* ≤ 0.10). Interestingly, cognitive ability did not predict self-rated entrepreneurial performance in this study.

**FIGURE 4:**
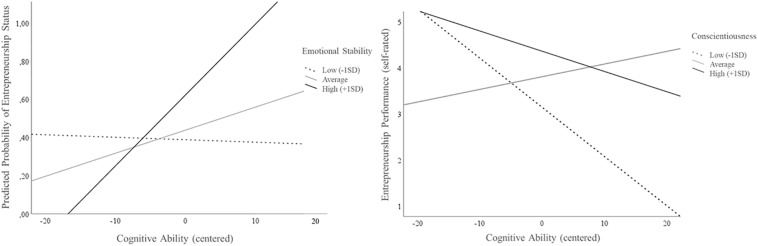
Relationship of cognitive ability to entrepreneurship status and success for three levels of emotional stability and conscientiousness.

To test the research question 3, it was studied whether the Big Five traits interact with cognitive ability when predicting entrepreneurial status and success. Interaction effects were studied using regression analyses which are summarized in Table 6. All predictors were centered around their means (Aiken and West, 1991) for these analyses. The results show significant interactions when predicting entrepreneurial status (Emotional Stability^∗^Ability: *Exp(B)* = 1.12, *p* ≤ 0.05, Δ*R*^2^ = 8%, *p* > 0.10) and entrepreneurial performance (Conscientiousness^∗^Ability: β = −0.28^∗^, *p* ≤ 0.05, Δ*R*^2^ = 7%, *p* > 0.10). Figure 4 graphs the interactions and shows that high emotional stability enhances the chance of becoming an entrepreneur in those with higher cognitive ability. Additionally, those who are already entrepreneurs perceive themselves as less performing when they are highly conscientious and clever. Concluding, the interaction assumption is partly supported.

### Brief Discussion of Study 3

Study 3 shows that the Big Five traits add novel information on whether a person successfully founds a business which cannot be derived from this person’s cognitive ability level. Additionally, more conscientious and (by trend) more emotionally stable entrepreneurs think that they perform better than less conscientious and stable colleagues. Notably, the current finding adds to existing research in two ways. First, it adds to the scant research on the importance of cognitive ability for becoming and performing as an entrepreneur. Second, it adds novel information as it shows that the Big Five traits add incremental validity over and partly interact with ability in the field of entrepreneurship. Therefore, it advances the psychology of entrepreneurship as suggested by Gorgievski and Stephan (2016). The fact that cognitive ability, the Big Five traits and their interaction explain little variance of a person’s entrepreneurial status and performance – even when considered conjointly – might be due to a generally strong influence of situational factors in entrepreneurship. For instance, political funding, a city’s infrastructure or current economic trends might be more impactful in entrepreneurship than in other contexts. Concluding, being smart is not necessarily enough for successfully launching and managing one’s own business. As shown by the current findings, traits like conscientiousness or emotional stability enhance the chance of successfully becoming and acting as an entrepreneur.

## General Discussion

This investigation examined whether socio-emotional skills add incremental validity over and interact with cognitive ability when predicting (1) the intention to become a business leader or entrepreneur, (2) the subsequent chance of actually becoming a top-level leader or entrepreneur, and (3) the success which is achieved by actual leaders or entrepreneurs. The socio-emotional skills we focus on are vocational interests and personality traits. Three main findings are highlighted. First, the vocational interest enterprising and social enhance the ability-based prediction of a person’s intention to become a leader or entrepreneur in the future. Social interests even interact with cognitive ability when predicting this intention (in the field of leadership). Second, the Big Five traits add incremental value beyond and can even interact with cognitive ability when predicting the position of leaders within the organizational hierarchy and the entrepreneurial status of a person. Finally, the success of actual leaders and entrepreneurs can be predicted by both, cognitive ability and the Big Five traits. Again, the latter add incremental validity beyond and interact with cognitive ability. Consequently, implications of these findings will be discussed in the light of the Leader–Trait–Emergence–Effectiveness model (LTEE; Judge et al., 2009) and the person–environment fit theory (Kristof, 1996).

### The Intention to Become a Leader or Entrepreneur Is Best Predicted by Considering Cognitive Ability and Vocational Interests Conjointly

Leadership and entrepreneurship intention are important prerequisites of becoming a leader or entrepreneur which, in turn, is a necessity for achieving leadership and entrepreneurship success (Judge et al., 2009). Our longitudinal findings are among the few to demonstrate that those with higher cognitive ability report higher intention to become a leader or entrepreneur. Even though this link is rather weak, it is in line with assumptions from the person–environment fit theory and shows that individuals with higher cognitive ability are more strongly drawn to jobs with higher complexity. Even more important, additionally considering a person’s enterprising and social interests adds considerable information to the question, who aims at becoming a leader or entrepreneur in the future. In fact, a person’s enterprising and social interests tell more about the future intentions than this person’s ability. More precisely, while higher enterprising interest enhance both, leadership and entrepreneurship intention, it is lower social interest that increase leadership aspiration. Remarkably, this finding of ours partly contradicts previous research which shows that social-oriented values rather predict higher leadership but lower entrepreneurship intention (Lechner et al., 2018). However, due to the young age of our study participants in Study 1 – they were on average only 17 years old – we argue that their vocational interests do not yet differentiate in such a strong manner as they might not be fully developed in this age group.

Interesting and anew is the finding that low social interests impede the intention to become a leader irrespective of this person’s cognitive ability whereas high social interests enhance this intention when the respective person shows high cognitive abilities. Thus, it obviously needs a certain interest level for developing the intention to take on a leading role or launch a business. What might be the mechanisms beyond this result? We assume that people with certain vocational interests seek out environments that fit their interests and thus more often find themselves in roles that match these. For instance, because enterprising individuals are interested in leadership and entrepreneurship duties, they seek out leadership and entrepreneurship roles more often. As shown by previous research, having more experience in such roles decreases personal reservations about being a leader or entrepreneur and in turn enhances the future leadership and entrepreneurship intention (Chan and Drasgow, 2001). In accordance with the trait activation theory (Tett and Guterman, 2000), we further argue that once individuals are presented with interest-matching situations, they more strongly express their interests and even develop them further. As such we speculate that once enterprising individuals get a first impression of what it is like being a leader or entrepreneur, their interest and intention for these jobs will further grow.

Based on the interaction between social interests and cognitive ability on leadership intention, we argue that those who appear clever enough to actually become leaders should get additional support to build up leadership-specific confidence, particularly when their social interest-level is only medium. If they are not supported it might be those with low social interests and low ability who most clearly express their intention to take on leading roles (and it is probably their intention which is heard best when potential leaders are needed). Importantly, this result demonstrates that higher social interests do not generally result in lower leadership intention (Bergner et al., 2019).

### Becoming a Top-Level Leader and Being Successful Is Best Predicted by Conjointly Considering Cognitive Ability and the Big Five Traits

Our results clearly show that a leader’s cognitive ability and the Big Five traits *conjointly* influence whether he/she reaches a top-level position and receives high income as well as good performance ratings. Notably, cognitive ability is linked to these criteria in a similar strength as the Big Five traits. According to Judge et al.’s (2009) LTEE model, we suppose that both cognitive ability and the Big Five traits are distal predictors and as such influence top-level positions and success not only in a direct manner but also in an indirect one via more proximal predictors. As we did not include proximal predictors, the question remains unanswered whether ability and the Big Five traits show a comparable indirect effect on success via such proximal predictors.

Importantly, our findings clearly demonstrate that being smart is not enough for becoming a top-level leader with high income and good performance ratings. In fact, a leader’s Big Five profile impacts his/her income, management level and performance rating independently of the leader’s cognitive ability. Our findings further show that personality traits can actually double the ability-based variance of top-level leadership emergence and success and therefore not only significantly but also meaningfully enhance their prediction. However, among the Big Five traits it is mainly extraversion, emotional stability and openness which are important. Some of these traits also interact with cognitive ability. For instance, openness facilitates the impact of ability on a leader’s income: high openness enhances a leader’s income only when this leader is rather smart. In contrast, emotional stability buffers the effect of ability on a leader’s income so that more neurotic and clever leaders report higher income than emotionally stable ones with a similar ability level. The same effect is found for top-level positions: leaders with similar cognitive ability end up in higher positions when they are more neurotic. Consequently, it might be argued that a certain level of ability is needed to boost the importance of openness and stability in leadership.

As previous research primarily focused on explaining the direct link between leadership success and *either* cognitive ability *or* the Big Five traits (e.g., Barrick et al., 2001; Judge et al., 2004), this study enriches literature by demonstrating their conjoined and interaction effect. In that regard it is important to keep in mind that working as a leader means dealing with cognitive and interpersonal complexity. While cognitive complexity arises for instance in situations where leaders have to deal with strategic decisions, interpersonal complexity can be observed when interacting with others, for instance in critical negotiations (Burke et al., 2006). To be most successful as a leader, cognitive and interpersonal complexity has to be managed and therefore cognitive ability and personality traits are required. This reasoning is in line with the person–environment fit theory (Kristof, 1996), which suggests that those whose abilities and personality traits meet the required tasks more likely complete the tasks successfully. As both are needed to successfully meet leadership tasks, the fit theory offers a valid explanation for why a person’s cognitive ability cannot compensate for this person’s Big Five traits.

### Becoming an Entrepreneur and Being Successful as Such Is Best Predicted by Conjointly Considering Cognitive Ability and the Big Five Traits

We reveal two important findings with regard to the impact of individual differences in entrepreneurship. First, a person’s cognitive ability may be used to predict who will found and manage a business. As this investigation is among few which test the direct link between cognitive ability and entrepreneurial status using in fact a validated ability measure, it can further be summarized that the impact of cognitive ability is rather small. Applying the LTEE model to the field of entrepreneurship, the inferior importance of cognitive ability might be due to the fact that it is a distal predictor of entrepreneurial status which unfolds its importance rather indirectly through more proximal predictors such as strategic thinking or recognizing business opportunities.

The second novel finding is that the Big Five traits contribute unique information to the question, who will found and manage his/her own business. In fact, it is solely the trait openness that improves the prediction of entrepreneurial status while the remaining Big Five traits seem less important in our study. Consequently, being smart is not enough for successfully founding a business but a person also needs to be open-minded, curious and fond of unconventional ideas and viewpoints (i.e., open). The same holds true for the entrepreneur’s self-perceived performance, which can be explained by his/her cognitive ability but is more precisely predicted when additionally considering the entrepreneur’s conscientiousness and emotional stability. With respect to the Big Five traits the results partly confirm a reoccurring Big Five profile for entrepreneurs. Even though not all effects became significant in the current study, the findings support an established profile which suggests that entrepreneurs compared to non-entrepreneurs show higher openness, conscientiousness, extraversion and stability, yet lower agreeableness (Obschonka and Stuetzer, 2017; Obschonka et al., 2017).

Importantly and anew, a person’s cognitive ability interacts with the Big Five traits when predicting his/her entrepreneurial status. The chance of successfully launching a business is highest for those with high emotional stability *and* cognitive ability. Neurotic individuals with similar ability have a smaller chance to successfully launch their venture. Cognitive ability also interacts with the Big Five traits when predicting entrepreneurship success. Our findings suggest that conscientiousness impedes the effect of ability on self-perceived success. Highly conscientious and smart individuals perceive themselves as less successful compared to those with an average level of conscientiousness. Consequently, it might be argued that a certain level of emotional stability is needed to boost the positive effect of cognitive ability on becoming an entrepreneur while being too conscientious impedes high (self-rated) entrepreneurial performance.

Even though previous meta-analyses clearly showed that the Big Five traits successfully differentiate between entrepreneurs and non-entrepreneurs (Brandstätter, 2011), the mechanisms beneath these findings are underexplored. We argue on the basis of the person–environment fit theory that becoming and successfully being an entrepreneur means dealing with cognitive complexity (e.g., analyzing market conditions, organizing the business) as well as interpersonal complexity (e.g., dealing with difficult customers, negotiating with deliverymen). To cope with both forms of complexity both – the cognitive ability level and the openness/conscientiousness level – have to be relatively high. In addition, we speculate that the Big Five traits enhance a person’s chance to found and successfully run a business due to his/her opportunity–recognition skills. Recognizing opportunities to make profit is essential for entre-preneurs as it directly influences venture performance (Sambasivan et al., 2009). According to the individual–opportunity nexus (Shane, 2007), certain traits enhance people’s chance of recognizing business opportunities and, because they do so, they are (1) more inclined to launch a business and (2) they are more successful entrepreneurs as they easily enlarge their product/service portfolio. On the grounds of the individual–opportunity nexus, we speculate that Big Five traits like openness enhance a person’s opportunity recognition skills and thus also indirectly influence entrepreneurial status and performance. We even extend this speculation to cognitive ability and argue that those with higher cognitive ability more likely recognize new business opportunities because they more easily “connect the dots” and see the same things they see every day with new eyes.

### Implication and Limitation

Our investigation has theoretical and practical implications. The most important theoretical implications are that being smart is not necessarily enough for aiming at a career in leadership or entrepreneurship, for emerging as top-level leader or entrepreneur and for receiving success as leader or entrepreneur. In fact, our findings reveal that there are some criteria – like entrepreneurship status – for which the impact of cognitive ability seems inferior. Second, our results imply that considering personality traits or vocational interests in addition to cognitive ability offers a more powerful prediction of success in leadership and entrepreneurship as well as leadership and entrepreneurship intention. From a theoretical point of view, these results support the person–environment fit theory for the leadership and entrepreneurship context and further extend the Leader–Trait–Emergence–Effectiveness model to the field of entrepreneurship. The third implication refers to the distinct importance of the different Big Five traits and vocational interests. Based on our findings, not all Big Five traits and vocational interests are equally important when increasing the ability-driven prediction. With regard to the Big Five traits, emotional stability, openness and conscientiousness are of particular importance for leadership/entrepreneurship emergence and success while enterprising is the most important vocational interest when predicting a person’s leadership or entrepreneurship intention. The final implication might also be the most insightful one. In an explorative manner it was shown that selected Big Five traits and vocational interests interact with cognitive ability when predicting leadership and entrepreneurship intention, emergence and success.

Practical implications of this investigation refer to personnel selection, development of leaders and entrepreneurs and career counseling for want-to-be leaders and entrepreneurs. Regarding personnel selection and development, the current findings clearly suggest considering personality aspects in addition to cognitive ones. Doing so should improve the prediction of who becomes successful and should further prevent from the circumstance that somebody is hired for ability but fired for personality. Moreover, when offering leader development programs or entrepreneurship education this investigation suggests including courses for the development of socio-emotional skills and not only focusing on knowledge-based skills. With regard to career counseling for want-to-be leaders and entrepreneurs, it is suggested to strengthen entrepreneurial interests, for instance, by exposing particularly those individuals to leadership and entrepreneurial tasks that show enterprising interests. By doing so they can deepen their interests which should then enhance their intention to take the lead or found a business in the future.

As with any study there are limitations to consider. First, the current findings refer to the most widely accepted personality model – the Big Five – but do not offer insights into which of the 30 Big Five sub-facets are particularly important. Therefore, future research should examine which sub-facets improve the ability-driven prediction of leadership and entrepreneurship emergence and success best. Moreover, it might be interesting to study whether other personality models like the HEXACO model or the Dark Triad also add incremental validity beyond cognitive ability in the field of leadership and entrepreneurship. Second, only one of the included studies uses longitudinal data. Continuing research clearly has to collect more longitudinal data to confirm the importance of socio-emotional skills beyond cognitive ability in a longer perspective. Worth mentioning is also that some outcomes in the current investigation were measured solely by self-perceptions, which are known to underlie certain biases that can result in more favorable ratings. Consequently, it might be interesting to examine a wider range and a more diverse set of outcomes, for instance, whether entrepreneurs fail or how their growth rate develops after several years. Doing so could provide better insights as the entrepreneurial sample would be more representative. Moreover, it might be worth examining the interaction effects between vocational interests/Big Five traits and cognitive ability in a more profound and theory-driven manner as this investigation offers only explorative insights. Finally, future research should use different ability measures, assess specific cognitive abilities (e.g., numerical intelligence) and refer to more profound ability tests for checking the stability of the current findings.

## Data Availability Statement

The datasets generated for this study are available on request to the corresponding author.

## Ethics Statement

The studies involving human participants were reviewed and approved by the University of Graz. The participants provided their written informed consent to participate in this study.

## Author Contributions

The author confirms being the sole contributor of this work and has approved it for publication.

## Conflict of Interest

The author declares that the research was conducted in the absence of any commercial or financial relationships that could be construed as a potential conflict of interest.

## Footnotes

1 The term leadership success or entrepreneurship success is used as an umbrella term and refers to both objective (e.g., income, venture growth) and subjective success criteria (e.g., performance-ratings).
